# Gypenosides Synergistically Reduce the Extracellular Matrix of Hepatic Stellate Cells and Ameliorate Hepatic Fibrosis in Mice

**DOI:** 10.3390/molecules28145448

**Published:** 2023-07-17

**Authors:** Han Li, Hanghang Wang, Aiping Yang, Mingzhen Xue, Junyang Wang, Qi Lv, Jian Liu, Lihong Hu, Yinan Zhang, Xiachang Wang

**Affiliations:** 1 Jiangsu Key Laboratory for Functional Substances of Chinese Medicine, Nanjing University of Chinese Medicine, Nanjing 210023, China; 2 Fujian Province Key Laboratory for the Development of Bioactive Material from Marine Algae, College of Oceanology and Food Science, Quanzhou Normal University, Quanzhou 362000, China

**Keywords:** gypenoside, liver fibrosis, PP2Cα, extracellular matrix, synergistic effect

## Abstract

Liver fibrosis resulting from chronic liver damage is becoming one of the major threats to health worldwide. Active saponin constituents isolated from *Gynostemma pentaphyllum* were found to possess a protective effect in liver diseases. Here, we obtained a naturally abundant gypenoside, XLVI, and evaluated its liver protection activity in both animal and cellular models. The results showed that it ameliorated acute and chronic liver injuries and lightened the process of fibrogenesis in vivo. XLVI can inhibit TGF-β-induced activation of hepatic stellate cells and ECM deposition in vitro. The underlying mechanism study verified that it upregulated the protein expression of protein phosphatase 2C alpha and strengthened the vitality of the phosphatase together with a PP2Cα agonist gypenoside NPLC0393. These results shed new light on the molecular mechanisms and the potential therapeutic function of the traditional herb *Gynostemma pentaphyllum* in the treatment of liver fibrosis.

## 1. Introduction

Liver fibrosis occurs in chronic liver injury, such as that caused by cholestasis, alcohol abuse, viral infection, drug abuse, and metabolic and autoimmune disorders, and may further lead to cirrhosis with portal hypertension, liver failure, and even liver carcinoma [1]. Liver fibrosis is the result of overproduction of the extracellular matrix (ECM). In this process, hepatocytes, Kupffer cells, and other inflammatory cells activate hepatic stellate cells (HSC) or even convert into myofibroblasts, breaking the dynamic synthesis and degradation equilibrium of ECM, including α-SMA and collagens. The TGF-β signaling pathway is the primary factor that drives fibrosis not only in the liver but also in other key organs. In HSC, the binding of TGF-β to its receptor TGFR phosphorylates Smad2 and Smad3 leads to their translocation to the nucleus with Smad4. The complex next initiates the transcription of profibrotic molecules and the tissue inhibitor of matrix metalloproteinases, resulting in myofibroblast activation and matrix deposition [2,3]. Although several anti-fibrotic compounds targeting TGF-β are now on the market and were verified to treat idiopathic pulmonary fibrosis and myelofibrosis [4], no effective treatment has yet been approved to treat liver fibrosis due to its complex pathology and diverse etiology [5,6].

*Gynostemma pentaphyllum* (Thunb.) Makino, also named Jiaogulan in Chinese, has been traditionally used as a medicinal herb in Eastern Asia for a long time. The crude extracts from *Gynostemma pentaphyllum* have multiple pharmacological effects, including hepatoprotective, anti-hyperlipidemia, and anti-inflammatory activities, but show no clear toxicity to animals [7]. Previous phytochemical studies on the genus *Gynostemma* found more than 300 saponins and sapogenins, most of which were identified as dammarane-type triterpenoids [8]. Our group, as well as other groups, previously demonstrated that different gypenosides had protective properties on liver injury-related animal models [9,10,11,12,13]. For example, we reported gypenoside NPLC0393 as the first small molecule agonist of PP2Cα to lessen TGF-β signal transduction in both canonical and non-canonical pathways [9,10]. NPLC0393 was also proven to significantly ameliorate the process of hepatic fibrosis by oral administration [11]. As part of a continuous effort to characterize triterpenes and explore their protective mechanisms, another gypenoside, XLVI, was characterized as a naturally abundant gypenoside [14] with an isolation yield of 3%. Since PP2Cα plays a vital role in the negative regulation of TGF-β signaling, we herein explored the protective effect of XLVI and its anti-liver fibrosis effects in vivo and in vitro. The underlying mechanism was shown to upregulate the RNA and protein levels of PP2Cα in hepatic stellate cells and to ameliorate carbon tetrachloride (CCl_4_)-induced hepatic fibrosis in mice. Furthermore, the study strikingly corroborated that XLVI and NPLC0393 synergistically inhibited hepatic stellate cell activation and reduced the release of ECM by emphasizing the vitality of this phosphatase.

## 2. Results

### 2.1. Protective Effects of XLVI on Acute and Chronic Liver Injuries In Vivo

**Figure 1:**
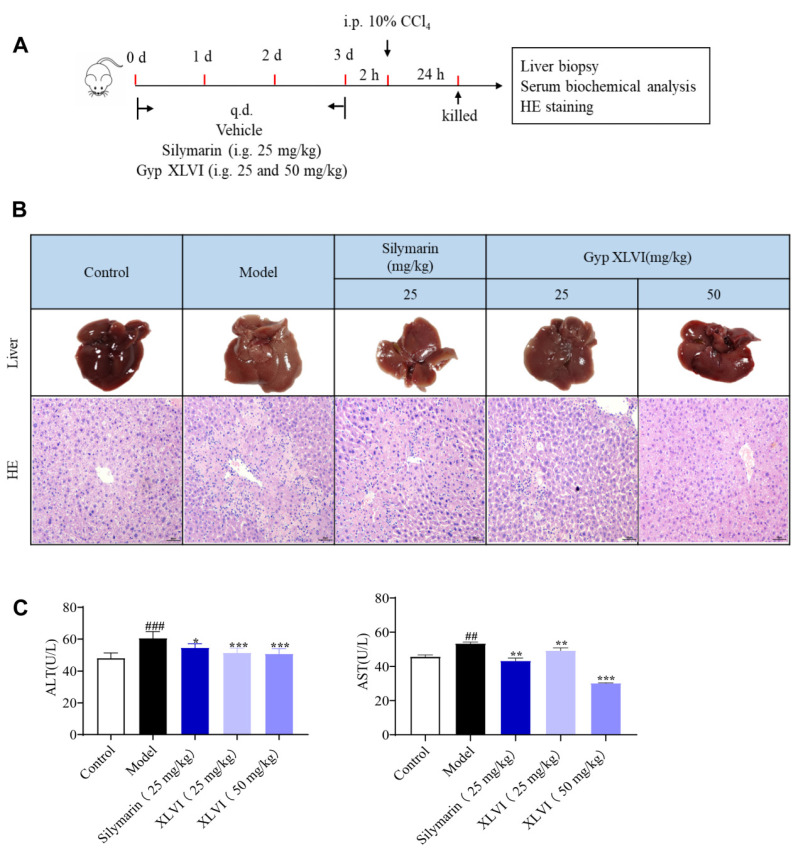
Protective effects of XLVI on CCl_4_ induced liver injury: (**A**) experimental model of acute liver injury; (**B**) representative images of intact livers and HE staining (20×) in each group; and (**C**) serum ALT, AST level (*n* = 8), ^##^ *p* < 0.01, ^###^ *p* < 0.001 versus Control group, * *p* <0.05. ** *p* < 0.01, *** *p* < 0.001 versus Model group. Data are presented as mean ± SD.

Previous studies have found that crude extracts from *G. pentaphyllum* have excellent therapeutic effects on liver fibrosis [11]. To clarify the active constituents in the total gypenoside components, one of the abundant gypenosides, XLVI was evaluated in vivo. A mice model of acute liver injury was successfully established after 24 h exposure to CCl_4_ (Figure 1A). Histopathological results indicated that CCl_4_ induced lipid accumulation, inflammatory cell infiltration, and widespread local cell death in liver tissue. In comparison with the CCl_4_ group, both XLVI (25 and 50 mg/kg) and silymarin (positive control, 25 mg/kg) treatment significantly decreased CCl_4_-induced liver pathological changes (Figure 1B). Both serum ALT and AST were increased significantly in the mice injected with CCl_4_, which were remarkably decreased by the administration of XLVI (Figure 1C). The above results indicated that XLVI had a significant therapeutic function on the acute liver injury model.

**Figure 2:**
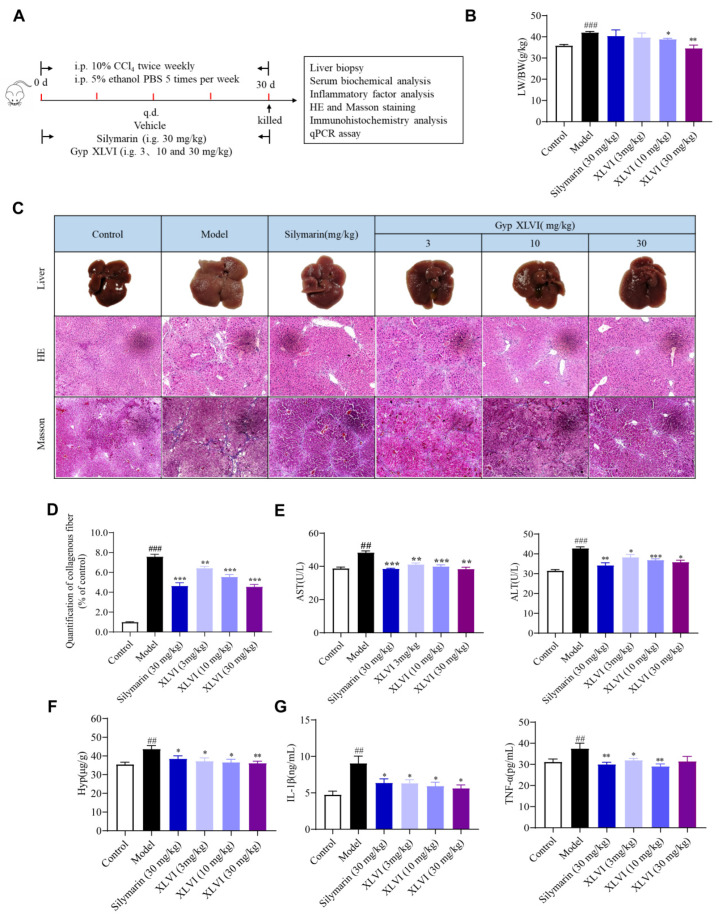
Effects of XLVI treatment on CCl_4_ and alcohol induced liver fibrosis: (**A**) establishment of liver fibrosis model; (**B**) liver weight/body weight ratio; (**C**) representative images of intact livers, HE, and Masson-staining in each group. The images were taken under a light microscope of 20 × magnification; (**D**) quantification of collagen fiber in Masson-stained sections; (**E**) levels of AST and ALT in serum; (**F**) HYP content in livers; and (**G**) IL-1β and TNF-α content in livers (*n* = 8), ^##^ *p* < 0.01, ^###^ *p* < 0.001 versus Control group, * *p* <0.05. ** *p* < 0.01, *** *p* < 0.001 versus Model group. Data are presented as mean ± SD.

Next, the chronic liver injury mice model was established to further evaluate the protective effect of XLVI (3, 10, and 30 mg/kg) in hepatic fibrogenesis. Due to the strong toxicity of CCl_4_, it was combined with alcohol to induce chronic liver injury as well as to reduce adverse reactions and mortality rates [15]. As depicted in Figure 2A, the livers collected from the CCl_4_ group were stiff and puffy, with an irregular and granular boundary, and the liver weight/body weight (LW/BW) ratio was significantly increased. The histopathological changes of HE staining showed disorder of the lobular architecture, steatosis of large number of hepatic cells, infiltration of inflammatory cells, and disturbance of the hepatic cord arrangement. In the XLVI and silymarin-treated groups, these symptoms were clearly mitigated (Figure 2B,C). The Masson’s trichrome-stained liver sections of each group revealed that there was a significantly increased fibroplasia area percentage in liver sections of the model group (Figure 2D), and that XLVI reversed the fibrotic area in a dose-dependent manner. Biochemical indexes showed that XLVI and silymarin reduced the levels of serum transaminase AST, ALT, and HYP at the same time (Figure 2E,F). In addition, XLVI also inhibited the upregulation of the typical inflammatory factors, such as TNF-α and IL-1β (Figure 2G). In summary, XLVI alleviated liver damage and reduced collagen deposition caused by long-term stimulation of CCl_4_ and alcohol in vivo.

### 2.2. XLVI Inhibits the Process of Fibrosis on the Chronic Liver Injury Mice Model

**Figure 3:**
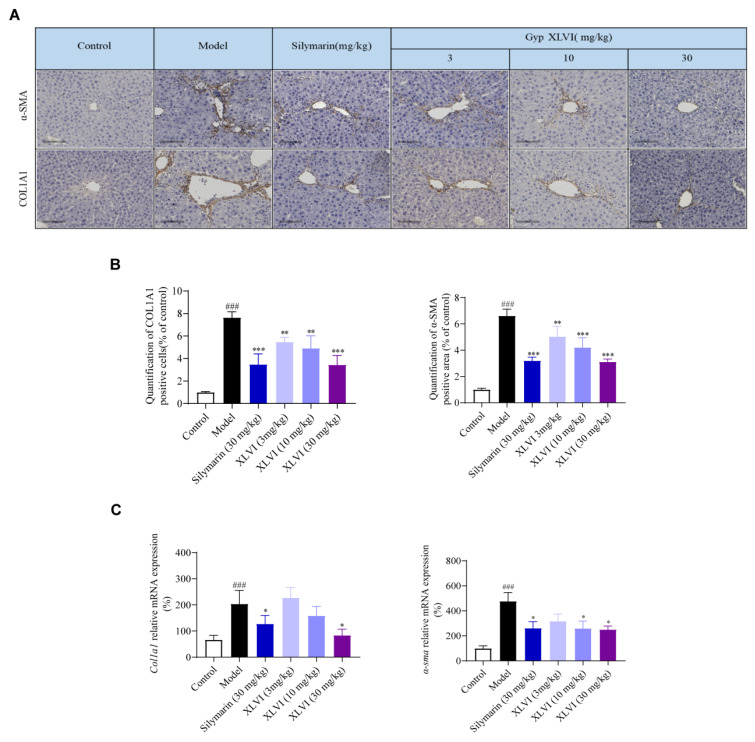
XLVI inhibits liver fibrosis in mice: (**A**) immunohistochemical results of α-SMA and COL1A1; (**B**) quantification of positive area of α-SMA and COL1A1; and (**C**) qPCR results of Col1a1 and α-Sma (*n* = 8). ^###^ *p* < 0.001 versus Control group, * *p* <0.05. ** *p* < 0.01, *** *p* < 0.001 versus Model group. Data are presented as mean ± SD.

Since α-SMA and COL1A1 are marked as key proteins in the activation of HSC and the development of fibrosis, we next examined their levels in the tissue collected from the chronic liver injury mice. Immunohistochemical results showed that the positive areas stained with α-SMA and COL1A1 antibodies were significantly increased in the model group compared with the blank group, which indicated that CCl_4_ and alcohol-induced injuries successfully promoted the expression of α-SMA and COL1A1 and the process of liver fibrosis. In the XLVI treatment groups, the deposition region of ECM was clearly reduced in a concentration-dependent manner (Figure 3A,B). qPCR analysis further verified that Col1a1 and α-Sma levels were inhibited in the liver tissues, especially in the high-dosage group treated with XLVI (Figure 3C).

### 2.3. XLVI Inhibited LX-2 Cells Activation via PP2Cα/P65/TGF-β Signal Pathway

**Figure 4:**
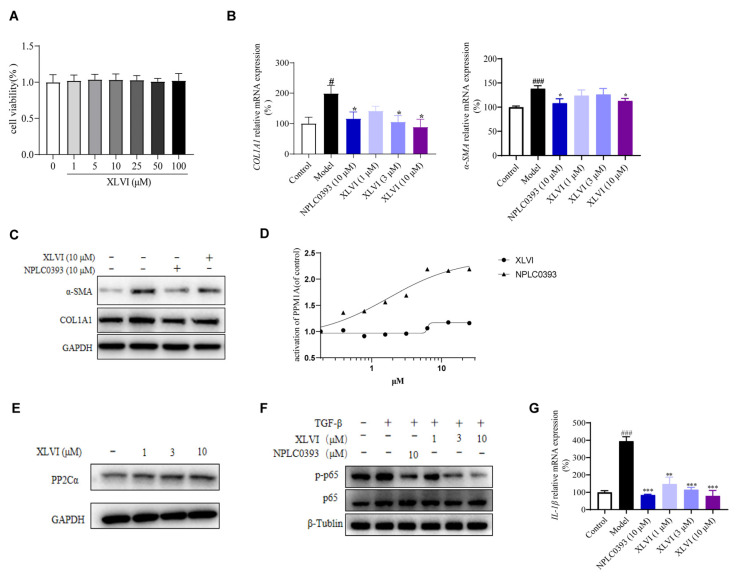
XLVI inhibited TGF-β1 stimulated LX-2 cells activation: (**A**) cell viability test; (**B**) expression of COL1A1 and α-SMA in LX2 cells was detected by Western blot analysis; (**C**) qPCR results of COL1A1 and α-SMA; (**D**) PP2Cα agonist activity of XLVI; (**E**) expression of PP2Cα in LX2 cells was detected by Western blot analysis; (**F**) expression of p65 and p-p65 in LX2 cells was detected by Western blot analysis; and (**G**) qPCR analysis of IL-1β. ^#^ *p* < 0.05, ^###^ *p* < 0.001 versus Control group, * *p* <0.05. ** *p* < 0.01. *** *p* < 0.001 versus Model group. Data are presented as mean ± SD.

It is understandable that the activation of quiescent HSCs leads to the transcription and secretion of pro-fibrogenic molecules, and to further differentiation into myofibroblasts [16]. The anti-fibrogenesis mechanism of XLVI on LX-2 HSC was then investigated. A CCK-8 assay revealed that it had little effect on LX-2 cell viability at concentrations ranging from 1 to 100 μM (Figure 4A). The mRNA levels of α-SMA and COL1A1 induced by the stimulation of TGF-β1 were effectively suppressed in the treatment group, in which XLVI at 10 μM showed a similar degree of effectiveness as the well-established HSC inactivator, gypenoside NPLC0393 [9,10,11]. The measurement of protein levels also verified the same trend found in the mRNA results (Figure 4B,C).

Our previous studies showed NPLC0393 acted as a PP2Cα agonist to dephosphorylate the nodal proteins in the TGF-β pathway that initiate the transcription of ECM components [9,10]. We suspected that XLVI, sharing the same dammarane scaffold as NPLC0393, may also agonize PP2Cα activity. Unfortunately, XLVI did not show agonistic activity on PP2Cα in the concentration range that inhibited ECM deposition compared to NPLC0393 (Figure 4D). However, we found that the cell treated with XLVI had significantly enhanced the expression of PP2Cα in a dose-dependent manner, which suggested that an increased protein amount may also suppress the downstream signaling pathway (Figure 4E). Since p65 phosphorylation is the key step in carrying out downstream fibrosis events in the HSC cells [13], we next examined its changes in the presence of XLVI. Although XLVI did not directly agonize PP2Cα activity, its promotion of PP2Cα expression level also inhibited the phosphorylation level of p65 without affecting the total amount of p65 protein in LX2 cells (Figure 4F). In addition, we found that XLVI and NPLC0393 can significantly suppress mRNA of pro-inflammatory cytokine IL-1β in HSC (Figure 4G). These findings indicated that XLVI inhibited the activation of HSC through the PP2Cα/p65/TGF-β signaling pathway.

### 2.4. XLVI and NPLC0393 had Synergistic Effect against the Activation of HSC Cells

**Figure 5:**
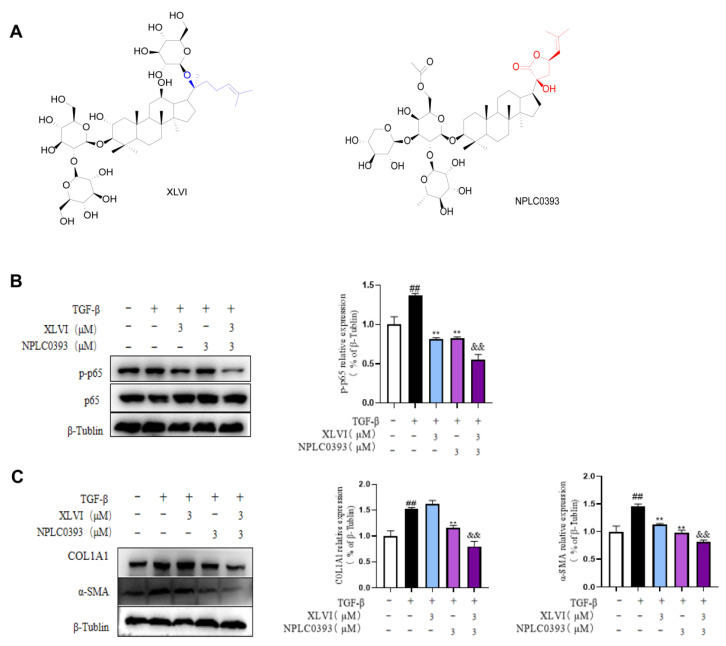
Gyps XLVI and NPLC0393 had synergistic effect against the activation of HSC cells: (**A**) chemical structures of gypenosides XLVI and NPLC0393; (**B**) expression of p65 and p-p65 in LX2 cells was detected by Western blot analysis; and (**C**) expression of COL1A1 and α-SMA in LX2 cells was detected by Western blot analysis. ^##^ *p* < 0.01 versus Control group, ** *p* < 0.01 versus Model group, ^&&^ *p* < 0.01 versus XLVI and NPLC0393 groups. Data are presented as mean ± SD.

Considering XLVI and NPLC0393 had complementary effects on PP2Cα proteins, we speculated that the combination of XLVI and NPLC0393 could have a better outcome in preventing HSC activation. A CCK-8 assay revealed that co-treatment of up to 20 μM did not show a viability change in the LX-2 cells. Western blot results showed that XLVI and NPLC0393 at 3 μM greatly reduced the phosphorylation of p65 and release of the ECM components COL1A1 and α-SMA (Figure 5B,C). The inhibitory effects were clearly more effective than single treatments with either compound. These results suggest that the dammarane components in *G. pentaphyllum* may prevent the development of liver fibrosis through synergistic mechanisms.

## 3. Discussion

The liver is the major organ responsible for detoxification, and the target organ for the metabolism of most toxic chemicals. Carbon tetrachloride (CCl_4)_ is known to cause liver toxicity, and the active metabolite of CCl_4_, trichloromethyl free radical (CCl_3_•) is mainly associated with CCl_4_-induced liver injury [17]. The acute liver injury model induced by CCl_4_ was selected to observe whether XLVI had a protective effect on the mice livers, with silymarin as a positive control. In the chronic hepatic fibrosis experiment, we used 0.5 mL/kg 10% CCl_4_ and 5% ethanol to decrease significant adverse effects and mortality. In the short-term and long-term experiments, XLVI displayed beneficial protective therapy against CCl_4_-induced liver injury and liver fibrosis. In this study, the effects of XLVI at 30 mg/kg were equal to, or even better than, the same dosage of silymarin as indicated by inflammation and fibrosis indicators. Liver fibrosis is characterized by various etiologies, high morbidity, and a complex pathogenesis, which cause the lack of appropriate treatments in clinics. The inhibition of HSC activation is a key step in the development of hepatic fibrosis. When the liver is damaged, multiple cytokines, such as TNF-α, TGF-β, and IL-1β, work together to promote HSCs activation and conversion into myofibroblasts, expressing α-SMA and secreting a large amount of ECM, especially collagen fibers (type I). Given that the TGF-β is recognized as the crucial factor in the progression of liver fibrosis, we selected 5 ng/mL TGF-β1 to stimulate LX-2 cells to explore the correlation between TGF-β1 and gypenosides. The qPCR and Western blot experimental results showed that XLVI can reduce TGF-β1-induced expression of α-SMA and COL1A1 in a dose-dependent manner.

PP2Cα is a member of the Ser/Thr protein phosphatase family [18] and has been identified as a negative regulator of several cellular signaling pathways, such as TGF-β and NF-κB [19]. We previously proved that gypenoside NPLC0393 inhibits the proliferation and activation of HSCs by directly agonizing PP2Cα [9]. Our study also found that NPLC0393 did not alter the protein expression and level of this phosphatase [10]. Following the same strategy, our current research shows that XLVI had a complementary effect on PP2Cα, in which it did not activate the enzyme but improved the expression in HSC cells, ultimately lowering phosphorylation of p65 and inhibiting TGF-β-induced activation of hepatic stellate cells. In addition, p65 is also the key regulator in NF-κB signaling, which is responsible for regulating the inflammatory response. When the NF-κB pathway is activated, it is translocated to the nucleus, where it mediates inflammatory liver injury by enabling the transcription of many pro-inflammatory genes such as TNF-α and IL-1β. The release of IL-1β from HSC leads to the secretion and deposition of ECM, which is crucial in HSC activation and liver fibrosis [20]. We also determined the IL-1β level in HSC treated with gypenosides (Figure 4), suggesting that dephosphorylation of p65 by the activation of PP2Cα clearly impeded the NF-κB related pro-inflammatory release.

The genus *Gynostemma* is widely used as traditional medicine in Eastern Asia for its broad biological activities in treatments for diabetes, hypertension, obesity, and hepatic protection. More than three hundred gypenosides were elucidated in the genus and a significant number of them showed notable pharmacological activities [8]. As the most distributed species, *G. pentaphyllum* is believed to be a surrogate resource of ginsenosides and other valuable dammarane-type natural products, due to its easy cultivation and short planting period [21]. Miscellaneous productions prepared from the plant as well as the crude extracts are included in the healthcare food list in China. Our group has worked on the active saponins for a long time and has isolated several gypenosides with liver protection effects [9,10,11]. However, varieties of *G. pentaphyllum* have changed the structure of gypenosides quite often since its cultivation origins, which makes it difficult to find abundant constituents in the species, and to also determine a specific activity with a single constituent. Gypenoside XLVI is an exceptional compound, with the high constituent content in the plant, collected from Zhejiang Province, confirmed by our group and other groups [22]. More importantly, we confirmed the synergistic protective effect in reducing fibrogenesis via the PP2Cα/TGF-β/ECM axis together with another gypenoside NPLC0393, suggesting a reasonable elucidation of the molecular mechanism of this traditional herb.

In summary, the liver protection activity of gypenoside XLVI was evaluated on both cellular and animal models. The results demonstrated that it’s able to alleviate chemical-induced liver injury and attenuated liver fibrosis through the inhibition of HSCs activation. The underlying mechanism suggests that the combination of gypenosides synergistically shut down the PP2Cα/TGF-β/p65 signaling pathway, thus highlighting the compounds as new therapeutic candidates for the treatment of liver fibrosis. The findings have encouraged us to identify and elucidate more pharmacological constituents in the traditional herb *G. pentaphyllum*, and further studies are now being conducted in our lab.

## 4. Materials and Methods

### 4.1. Materials

XLVI (purity > 98.0%) and NPLC0393 (purity > 98.0%) were provided by Jiangsu Key Laboratory for Functional Substances of Chinese Medicine (Nanjing, China). Silymarin, CCK-8 kit, 0.25% trypsin, phenylmethylsulfonyl fluoride (PMSF), and NP-40 lysis buffer were obtained from Liangwei Biotechnology Co., Ltd. (Nanjing, China). The human LX2 cells were purchased from Mingzhoubio Company (No. MZ-0286). Fetal bovine serum (FBS), DMEM medium, penicillin-streptomycin (P/S) and phosphate-buffered saline (PBS) were obtained from Gibco Company (Grand Island, VT, USA). TGF-β1, antibodies recognizing COL1A1, α-SMA, p65, p-p65 (Ser536), GAPDH, and β-tubulin were obtained from Cell Signaling Technology (Danvers, MA, USA). Mouse secondary antibodies and rabbit secondary antibodies were provided by Signalway Antibody (College Park, MD, USA). The primers for *COL1A1*, *α-SMA*, and *IL-1β* were designed and synthesized by Shanghai Sangon Biological Engineering Co., Ltd. (Shanghai, China). ALT, AST, HYP, IL-1β, and TNF-α ELISA kits were purchased from Nanjing Jiancheng Bioengineering Institute (Nanjing, China). RNase Mini Kits, Trizol reagent, and HiScript II Q RT SuperMix for qPCR were obtained from Vazyme Biotech Company (Nanjing, China).

### 4.2. LX-2 Cell Culture

The human LX2 cells were cultured in a DMEM medium with 1% P/S and 10% FBS. Cells were cultured at 37 °C in a humidified atmosphere containing 5% CO_2_.

### 4.3. Cell Proliferation Analysis

The LX2 cells were inoculated in a logarithmic growth phase into 96 well plates. Cells were then treated with different concentrations of XLVI for 24 h. A CCK-8 solution (10 μL) was added to the stained cells for another 2 h. A microplate reader (Infinite M Nano, Nanjing, China) at 450 nm was used to measure the absorbance.

### 4.4. Western Blot Assay

Total protein was extracted from LX2 cells with NP-40 lysis buffer which contained a phosphatase inhibitor cocktail (1: 100) and protease inhibitor PMSF (1: 100). After quantification by the BCA assay (Biyuntian Biotechnology, Shanghai, China), the equal protein was separated using sulfate-polyacrylamide gel electrophoresis, then transferred to the polyvinylidene fluoride membrane. Then the membrane was blocked by 5% skim milk for 1 h at room temperature and incubated with the indicated primary antibody, including COL1A1, α-SMA, p65, p-p65, GAPDH, and β-tubulin, then incubated with HRP-conjugated secondary antibodies. The corresponding bands were visualized by electrogenerated chemiluminescence (ECL). Immunoblot intensities were quantified by densitometric analysis (Image J, rsb.info.-nih.gov/ij).

### 4.5. Animals and Groups of CCl4-Induced Liver Injury Experiment

The 6–8 weeks-old male mice C57BL/6 were supplied by Jiangsu Jicuiyaokang Animal Cultivation Farm (Nanjing, China). All animal experimental procedures were approved by the Animal Care Committee of the Nanjing University of Chinese Medicine.

The mice were divided into five subgroups randomly (six mice per group): a control group, model group, silymarin group (25 mg/kg), and XLVI groups (25 and 50 mg/kg). Mice were given corresponding drug treatment once a day for three consecutive days except for the control and model groups. Two hours after the last administration, the mice were intraperitoneally injected with 0.1 mL/10 g 10% CCl_4_ solution (olive oil:CCl_4_ = 9:1 dilution) intraperitoneally except for the control group.

### 4.6. Animals and Groups of CCl_4_ and Ethanol-Induced Liver Fibrosis Experiment

Forty-eight C57/BL6 male mice were obtained in the same manner as above. The mice were divided into six groups randomly (eight mice per group): a control group, model group, silymarin group (30 mg/kg), and low-, medium- and high-dosage XLVI groups (3, 10, and 30 mg/kg). The liver fibrosis model was generated by alternating intraperitoneal injections of 0.5 mL/kg 10% CCl_4_ in olive oil (1:10) twice per week, and an isovolumetric dose of 5% ethanol in PBS five times per week. Silymarin and XLVI in 0.5% CMC-Na were administered intra-gastrically, respectively. After four weeks, overnight-starved mice were executed 48 h after the last CCl_4_ injection.

### 4.7. Blood and Tissue Samples Preparation

At the end of the above experiment, mice were anesthetized with isoflurane, weighed, and their blood was collected from the retro-orbital venous plexus. Serum samples were extracted and stored at −20 °C. Mice were then euthanized by cervical dislocation, the liver samples were cut, rinsed in ice-cold PBS, dried, and then weighed. The liver index was calculated as: liver index = liver weight/body weight × 100%. Each left liver lobe was cut and stored in 10% buffered neutral formalin for further immunohistochemical and histopathological analysis. The remaining portion was preserved at −80 °C for subsequent biochemical studies and qPCR analyses.

### 4.8. Histological Evaluation

Pathological staining was commissioned to Liangwei Biotechnology Co., Ltd. (Nanjing, China). After fixation in 10% buffered neutral formalin for 24 h, the liver tissues of each group were consistently processed as paraffin slices. Specifically, the livers were rinsed with water, dehydrated in ethanol, cleared in xylene, and then embedded in paraffin. Paraffin blocks were cut as 4 to 5 μM thick slices. After being mounted on glass slides, the tissue slices were deparaffinized and stained with HE or Masson-staining. The histological damage or fibrosis was examined under a microscope (Leica, Tokyo, Japan). By using Image-Pro Plus 7.0 analytical software (Media Cybernetics, Rockville, MD, USA), the fibrous tissues of Masson-stained regions were quantified for their area percentage.

### 4.9. Immunohistochemistry Analysis

After being dewaxed sequentially with xylene and gradient alcohol, the paraffin sections were immersed in a citrate-repair solution, then heated for antigen repair in a microwave. After rinsing three times with PBSr, the sections were supplemented with an endogenous peroxidase blocker and incubated for ten min at room temperature. Again, they were rinsed with PBS three times (three min each) and incubated with goat-serum working solution for 15 min at room temperature. Then, COL1A1 (1: 500) or α-SMA (1: 500) were added and the sections were incubated for 1 h at 37 °C. After rinsing with PBS three times (three min each), a biotin-labeled second antibody was added, and sections were incubated for a further 15 min at room temperature. After being rinsed again with PBS (three min for three times), the sections were incubated with a horseradish enzyme-labeled streptavidin working solution for another 15 min. Again, the sections were rinsed for three min with PBS (three times), then stained for 25 s with a DAB chromogenic and stopped by using PBS. The sections were then stained with hematoxylin for 90 s, differentiated by hydrochloric acid alcohol for 10 s, rinsed in water to turn blue for 10 min, dehydrated by gradient alcohol, transparentized by xylene and sealed using a neutral gum. Image-Pro Plus 7.0 analytical software (Media Cybernetics, Rockville, MD, USA) was used to select eight samples in each group for COL1A1- and α-SMA-positive area quantification.

### 4.10. ELISA

The serum samples obtained were removed from −80 °C freezer and placed at room temperature to allow for defrosting. The determination of serum ALT and AST levels was performed in strict accordance with the ELISA kit instructions. For each mouse, 30 mg of liver tissue was taken and cleaned with PBS. After drying with tissue paper, 270 μL PBS solution was added and transformed into tissue homogenate with the help of a multi-sample tissue grinder. HYP, IL-1β, and TNF-α contents were then measured.

### 4.11. qPCR Assay

Total RNA was extracted using Trizol and reverse-transcribed into single-stranded cDNA using Hiscript Q RT Supermix (Vazyme, Nanjing, China). RT qPCR involves SYBR green-based assays run using the LightCycler 96 system. The data were standardized to Actb expression in each sample. The specific primer pairs in the experiment are summarized in Table 1.

For each mouse, 20 mg of liver tissue was taken and cleaned with PBS. They were dried with tissue paper, to which 180 μL RNA lysate was added, and were then transformed into tissue homogenate using a multi-sample tissue grinder. For qPCR experiment, the used primer sequences are listed in Table 1. The internal control was β-actin, and the expression of relative mRNA expression was calculated according to the 2^−ΔΔCt^ method.

### 4.12. PP2Cα Kinase Activity Test

The effect of XLVI on PP2Cα dephosphorylation of p-nitrophenyl phosphate (pNPP, SolarBio, Beijing, China) was determined by a continuous assay. PP2Cα (10 µg/mL) was incubated with gypenosides at room temperature for 1.5 h, with 1% DMSO as control. Reactions were started by the addition of 4 mM pNPP, and the absorbance change was then monitored at 410 nm.

### 4.13. Statistical Analysis

The plots were constructed by GraphPad Prism 8.0 (GraphPad Software, Inc., San Diego, CA, USA) and the statistical analysis was performed with the SPSS 19.0 software (SPSS, Chicago, IL, USA). The values were expressed as means ± standard deviation (SD). One-way and two-way ANOVA were applied for comparisons between groups, and *p* < 0.05 was indicated as statistically significant.

### 4.14. Ethics Statement

The Nanjing University of Chinese Medicine Experimental Animal Ethics Committee ratified all animals in this study [Permission No. SYXK(SU)2018-0049, 2020.10.11]. Procedures and protocols were performed in strict accordance with The Care and Use of Laboratory Animals, published by The United States National Institutes of Health (NIH Publication, No. 8023, revised 1978).

## Figures and Tables

**Table 1:** The primer sequences used in the qPCR experiment.

Species	Genes	Primer sequences
HUMAN	α-SMA	Forward:5′-AAAAGACAGCTACGTGGGTGA-3′
		Reverse: 5′-GCCATGTTCTATCGGGTACTTC-3′
	COL1A1	Forward:5′-GAGGGCCAAGACGAAGACATC-3′
		Reverse: 5′-CAGATCACGTCATCGCACAAC-3′
	IL-1β	Forward:5′-TTCGACACATGGGATAACGAGG-3′
		Reverse: 5′-TTTTTGCTGTGAGTCCCGGAG-3′
	β-actin	Forward:5′-CTCCATCCTGGCCTCGCTGT-3′
		Reverse: 5′-GCTGTCACCTTCACCGTTCC-3′
MOUSE	α-SMA	Forward:5′ GTCCCAGACATCAGGGAGTAA-3′
		Reverse: 5′-TCGGATACTTCAGCGTCAGGA-3′
	COL1A1	Forward:5′-GCTCCTCTTAGGGGCCACT-3′
		Reverse: 5′-CCACGTCTCACCATTGGGG-3′
	IL-1β	Forward:5′ GAAATGCCACCTTTTGACAGTG-3′
		Reverse: 5′-TGGATGCTCTCATCAGGACAG-3′
	β-actin	Forward:5′-CGTTGACATCCGTAAAGACC-3′
		Reverse: 5′-AACAGTCCGCCTAGAAGCAC-3′

## Data Availability

Not applicable.
